# Supplementary material for: A molecular barcode and web-based data analysis tool to identify imported Plasmodium vivax malaria
Source: Commun Biol. 2022 Dec 23;5:1411. doi: 10.1038/s42003-022-04352-2 (PMC9789135; doi:10.1038/s42003-022-04352-2)
Supplement: Supplementary file 2 — Supplementary Information [file 42003_2022_4352_MOESM2_ESM.pdf]

Supplementary Information file for:

A molecular barcode and web-based data analysis tool to identify imported *Plasmodium vivax* malaria

Hidayat Trimarsanto[1,2], Roberto Amato[3], Richard D Pearson[3], Edwin Sutanto[2,4], Rintis Noviyanti[2], Leily Trianty[2], Jutta Marfurt[1], Zuleima Pava[1], Diego F Echeverry,[5,6,7], Tatiana M Lopera-Mesa[8], Lidia M Montenegro[8], Alberto Tobon-Castano[8], Matthew J Grigg[1,9], Bridget Barber[1,9], Timothy William[9,10], Nicholas M Anstey[1], Sisay Getachew[11,12], Beyene Petros[11], Abraham Aseffa[12], Ashenafi Assefa[13], Awab G Rahim[14,15], Nguyen H Chau[16], Tran T Hien[16], Mohammad S Alam[17], Wasif A Khan[17], Benedikt Ley[1], Kamala Thriemer[1], Sonam Wangchuck[18], Yaghoob Hamedi[19], Ishag Adam[20], Yaobao Liu[21,22], Qi Gao[21], Kanlaya Sriprawat[23], Marcelo U Ferreira[24,25], Moses Laman[26], Alyssa Barry[27,28,29], Ivo Mueller[28,30], Marcus V G Lacerda[31,32], Alejandro Llanos-Cuentas[33], Srivicha Krudsood[34], Chanthap Lon[35], Rezika Mohammed[36], Daniel Yilma[37], Dhelio Batista Pereira[38], Fe E J Espino[39], Cindy S Chu[14,23], Ivan D Velez[8], Chayadol Namaik-larp[40], Maria F Villegas[41], Justin A Green[42], Gavin Koh[42], Julian C. Rayner[3,43], Eleanor Drury[3], Sónia Gonçalves[3], Victoria Simpson[3], Olivo Miotto[3,14], Alistair Miles[3], Nicholas J White[14,44], Francois Nosten[23,44], Dominic P Kwiatkowski[3], Ric N Price[1,14,44], Sarah Auburn[1,14,44*]

1 Global and Tropical Health Division, Menzies School of Health Research and Charles Darwin University, Darwin, Northern Territory, Australia

2 Eijkman Institute for Molecular Biology, Jakarta, Indonesia

3 Wellcome Sanger Institute, Wellcome Genome Campus, Cambridge, United Kingdom

4 Exeins Health Initiative, Jakarta, Indonesia

5 International Training and Medical Research Center (CIDEIM), Cali, Colombia

6 Departamento de Microbiología, Universidad del Valle, Calle 4b No. 36-00 Edificio 120, Oficina 332, Cali, Colombia

7 Universidad Icesi, Calle 18 No. 122-135, Cali, Colombia

8 Malaria Group, Universidad de Antioquia, Medellin, Colombia

9 Infectious Diseases Society Sabah-Menzies School of Health Research Clinical Research Unit, Kota Kinabalu, Sabah, Malaysia

10 Clinical Research Centre, Queen Elizabeth Hospital, Sabah, Malaysia

11 College of Natural Sciences, Addis Ababa University, Addis Ababa, Ethiopia

12 Armauer Hansen Research Institute, Addis Ababa, Ethiopia

13 Ethiopian Public Health Institute, Addis Ababa, Ethiopia

14 Mahidol-Oxford Tropical Medicine Research Unit, Mahidol University, Bangkok, Thailand

15 Nangarhar Medical Faculty, Nangarhar University, Ministry of Higher Education, Jalalabad, Afghanistan

16 Oxford University Clinical Research Unit, Hospital for Tropical Diseases, Ho Chi Minh City, Vietnam

17 Infectious Diseases Division, International Centre for Diarrheal Diseases Research, Bangladesh Mohakhali, Dhaka, Bangladesh

18 Royal Center for Disease Control, Department of Public Health, Ministry of Health, Thimphu, Bhutan

19 Infectious and Tropical Diseases Research Center, Hormozgan University of Medical Sciences, Bandar Abbas, Hormozgan Province, Iran

20 Faculty of Medicine, University of Khartoum, Khartoum, Sudan

21 National Health Commission Key Laboratory of Parasitic Disease Control and Prevention, Jiangsu Provincial Key Laboratory on Parasite and Vector Control Technology, Jiangsu Institute of Parasitic Diseases, Wuxi, China
22 School of Public Health, Nanjing Medical University, Nanjing, China
23 Shoklo Malaria Research Unit, Mahidol-Oxford Tropical Medicine Research Unit, Faculty of Tropical Medicine, Mahidol University, Mae Sot, Thailand
24 Department of Parasitology, Institute of Biomedical Sciences, University of São Paulo, São Paulo, Brazil
25 Global Health and Tropical Medicine, Institute of Hygiene and Tropical Medicine, NOVA University of Lisbon, Lisbon, Portugal
26 Papua New Guinea Institute of Medical Research, Madang, Papua New Guinea
27 Deakin University, Victoria, Australia
28 Population Health and Immunity Division, The Walter and Eliza Hall Institute of Medical Research, Victoria, Australia
29 Department of Medical Biology, The University of Melbourne, Parkville, VIC, Australia
30 Department of Parasites and Insect Vectors, Institut Pasteur, Paris, France
31 Fundação de Medicina Tropical, Manaus, Brazil
32 Fundação Oswaldo Cruz, Manguinhos, Rio de Janeiro, Brazil
33 Universidad Peruana Cayetano Heredia, Lima, Peru
34 Mahidol University, Bangkok, Thailand
35 Armed Forces Research Institute of Medical Sciences, Bangkok, Thailand
36 University of Gondar, Gondar, Ethiopia
37 Jimma University, Jimma, Ethiopia
38 Centro de Pesquisa em Medicina Tropical, Porto Velho, Brazil
39 Research Institute for Tropical Medicine, Manilla, Philippines
40 Umphang Hospital, Tak, Thailand
41 Centro de Investigaciones Clinicas, Cali, Colombia
42 GlaxoSmithKline, Brentford, United Kingdom
43 Cambridge Institute for Medical Research, School of Clinical Medicine, University of Cambridge, Cambridge, United Kingdom
44 Centre for Tropical Medicine and Global Health, Nuffield Department of Medicine, University of Oxford, Oxford, United Kingdom

*Corresponding author: Dr Sarah Auburn, sarah.auburn@menzies.edu.au, Menzies School of Health Research, PO Box 41096, Casuarina, Darwin, NT 0811, Australia; Tel: (+61) 8 8946 8503

Table of Contents

Supplementary Methods....4
Analysis Overview....4
Dataset....4
Data Preparation....4
Development of Bi-Allele Likelihood (BALK) Classifier and Comparative Analysis with Bernoulli Naive Bayes....6
Overview of required classifier....6
Overview of Naive Bayes classifier....7
Classifying biallelic SNP data with Bernoulli Naive Bayes (BNB)....8
Classifying with Biallele Likelihood (BALK) classifier....8
Evaluating the performance of BNB and BALK classifiers....8
SNP Selection....10
SNP selection using DecisionTree....10
SNP selection by F_{ST}....11
Evaluation and Assessment....14
Comparative assessment of candidate SNP panels....14
Assessment of the durabililty of candidate SNP panels....14
Validation using independent dataset....15
Web-based Data Analysis Platform for End-Users....15
Implementation and Web Service Availability....15
Supplementary Tables....16
Supplementary Figures....22
Supplementary References....27

Supplementary Methods

Analysis Overview

An overview of the analysis plan for this study is outlined as a flowchart presented in **Figure 1** of the main text. In brief, the analytical process involved three key steps:

1. Data preparation

   This step entailed a series of sample and SNP filtering steps to obtain suitable genomic datasets for downstream analysis. The filtering steps aimed to derive an optimal balance of maximum numbers of samples and SNPs against minimum genotype missingness (i.e., positions with no reads).

2. SNP selection

   This step entailed selection of SNPs using DecisionTree and HFST approaches to obtain panels with the minimal numbers of SNPs that could effectively classify infections by country at the different thresholds applied using the classifier developed in this study.

3. Evaluation and assessment

   This step entailed evaluation of the selected SNP sets and the classifiers with stratified k-fold cross-validation. In addition to evaluation with no missing data, the performance of the selected SNP sets was further assessed at varying levels of missing data (i.e., genotype failures). Lastly, the performance of the selected SNP sets was assessed using an independent dataset.

An online, open-access platform was then developed for end-users to readily analyze data generated at the selected SNP sets to predict the country of origin.

Dataset

The dataset used in the analysis included genomic data on P. vivax isolates derived from the Malaria Genomic Epidemiology (MalariaGEN) P. vivax Genome Variation Project **Pv4 open dataset**, which were collected from 26 countries[1]. All samples were processed within the framework of the MalariaGEN P. vivax Genome Variation Project. Briefly, all new isolates were subjected to whole genome sequencing at the Wellcome Sanger Institute using standard library preparation and sequencing on the Illumina HiSeq platform to generate 150bp paired end reads. The new data was combined with publicly available data, aligned against the P. vivax PvP01 reference[2] and subjected to variant calling and annotation according to best practices in the MalariaGEN framework[1]. The resultant VCF files, as well as the accompanying metadata for the Pv4 open dataset are available in the MalariaGEN repository (https://www.malariagen.net/resource/30).

For the analysis in this study, the dataset was divided into two parts, the **training set** and the **validation set**. The training set consisted of all Pv4 isolates, excluding a set of samples from the GSK study that were reserved for the validation set. Specifically, the validation set consisted of all isolates from the GSK study except for those from the Philippines. The decision to use the specified GSK study samples for validation was based on convenience owing to the later timing at which sequencing data from the GSK study were made available.

Data Preparation

An overview of the data preparation steps is outlined in section *a. Data Preparation* of the flowchart presented in **Figure 1**.

Prior to data filtering, all Pv4 VCF files from the MalariaGEN repository were reprocessed to set a minimum read depth of 1. Instead of using the GT field, individual genotype calls were redefined as heterozygotes based on an arbitrary threshold of a minor allele read ratio > 0.1 and a minimum of 2 reads for each allele; all other genotype calls were defined as homozygous for the major allele. All VCF processes were performed using BCFTools and custom python scripts. All consecutive analysis used the genetic data from this reprocessed VCF file.

The training dataset was filtered initially to exclude recurrent infections in the same individuals based on the Pv4 metadata information and to include only samples to which we had access (data published or provided with permission from the sample contributors as our study was undertaken prior to the open data release). All samples whose countries were represented by less than 4 genomic samples (arbitrary threshold) were also removed. In total, this filtering process resulted in an **initial dataset** comprising 1,348 samples as presented in **Supplementary Table 1**, from 21 countries as shown in **Supplementary Figure 1**. With this dataset, from the initial 2,671,112 discovered variants provided in Pv4, we derived a set of 662,641 high-quality bi-allelic SNPs with VQSLOD score > 0 and minimum Minor Allele Count (MAC) of 2 by employing BCFTools. This dataset with 1,348 samples and 662,641 was denoted as **Dataset 0**.

Dataset 0 was then subjected to further filtering to remove non-independent samples, arbitrarily defined as isolate pairs with genetic distance less than 0.001 (0.1%). For this and consecutive analysis, genetic distances were computed as proportion of nucleotide or allele differences, with heterozygous calls considered as identical to homozygote calls and any missing genotype was omitted from the calculation. Amongst non-independent sample pairs, the isolate with the higher percentage of genotype failures was removed from the dataset; this removal process was iterated until all non-independent isolates had been removed from the dataset. This filtering process employed a custom Python script and resulted in 1,227 samples with 662,641 SNPs denoted as **Dataset 1**. The following pseudocode illustrates this filtering process:

```
Prepare dataset X
Calculate pairwise genetic distance based on proportion of allele differences
WHILE there are pairs with genetic distance < 0.001
        Get the pair with lowest genetic distance
        Remove the isolate of the pair with higher missingness from dataset X
```

Dataset 1 was then subjected to iterative data quality filtering by removing samples and SNPs to obtain the best representative number of samples and informative SNPs, designated as SNPs with MAC ≥ 2, with no genotype missingness. The following pseudocode illustrates the process of obtaining the optimal number of samples and informative SNPs:

```
Prepare dataset X
Calculate sample missingness of dataset X
Sort sample set in descending order of missingness
REPEAT
        Calculate the number of informative SNPs without any missingness in dataset X
        Collect the number of samples and number of informative SNPs above
        Remove sample with highest missingness from sample set and dataset X
UNTIL no more sample in sample set and dataset X
Plot the collected number of samples vs the number of informative SNPs
Plot the collected number of samples vs the changes of the number of informative SNPs
```

The rationale to perform the above filtering process is that while removing relatively lower quality samples from the dataset will increase the number of SNPs without any missing genotype data, the sample removal process might decrease the number of informative (or non-monomorphic) SNPs. **Supplementary Figure 2** provides a plot of the number of filtered samples against the number of filtered SNPs and the differences in the number of SNPs (delta SNPs). Based on this plot, with informative SNPs defined as SNPs with MAC ≥ 2, we identified 826 samples and 229,317 SNPs as the optimum for inclusion in **Dataset 2**.

The isolates in Dataset 2 were initially assigned the country in which the patient presented at the clinic with the infection based on the Pv4 metadata. This initial country-level assignment was evaluated further using 1) country-level prediction derived from the genome-wide data classification with the BALK classifier (described in the next section) and 2) manual confirmation by constructing a neighbor-joining tree. Briefly, the BALK classifier was trained with the Dataset 1 against all 229,317 SNPs, and then each isolate of the Dataset 2 was classified by the trained BALK. For the manual confirmation using neighbor-joining tree, a genetic distance matrix was computed from Dataset 1 and a neighbor-joining tree was constructed using the Ape R library [3]. Isolates whose country assignment differed from the prediction result of the trained BALK classifier and that were not in the same country cluster as observed manually from the neighbor-joining tree were considered suspected imported infections and removed from the dataset to produce **Dataset 3**, comprising 799 samples and 229,317 SNPs.

For comparative assessment of candidate SNP panels, a new dataset (**Dataset 4**) was produced which comprised the samples in Dataset 3, but only with the SNPs selected by the consecutive SNP selection process and the Broad barcode panel; a SNP barcode panel which consisted of 42 SNPs that had been developed by the Broad Institute team to fingerprint *Plasmodium vivax* and which has already been implemented in several studies[4-6]. Whilst the original Broad barcode comprises 42 SNPs, we were only able to use 38 of these SNPs, herein referred to as **BR38**, since 4 SNPs were either non-biallelic within the Pv4 dataset or could not be genotyped using amplicon sequencing (i.e., were non-assayable)[4].

A similar filtering process was applied to the validation set (comprising all the GSK study samples except those from the Philippines). Recurrent infections, inferred from the Pv4 metadata, were also removed from the validation set. However, instead of obtaining the optimal number of samples and informative SNPs as for the training set, we directly included the same 229,317 SNPs used in the training set. Any non-independent samples were then removed using the same 0.001 threshold of genetic distance, using a similar procedure to that applied to the training set. Country-level assignment was assessed using the same trained BALK classifier as the training set, and a neighbor-joining tree was constructed by combining with Dataset 3 for manual confirmation, resulting in 142 samples in the validation set. **Supplementary Figure 3** presents a neighbor-joining tree generated on Dataset 3 combined with the 142 validation samples using the 229,317 SNPs. Further SNP filtering was then applied to only include the 38-SNP Broad barcode (BR38) and the SNPs selected by the SNP selection process, producing the **Independent Validation Dataset**.

Development of Bi-Allele Likelihood (BALK) Classifier and Comparative Analysis with Bernoulli Naive Bayes

Overview of required classifier

In the machine learning field, a classifier is an algorithm that can be used to assign a *class* label to a *data input* based on a set of specific *features*. In this study, for the purpose of identifying the country of origin of a sample (essentially a *P. vivax* genome), the *class* is the country of origin, the *features* are a set (or panel) of SNP positions (markers), and the *data input* is the allele at each SNP position. This section describes the processes underlying the decision to apply the Bi-Allele Likelihood Classifier in our study (i.e., as the vivaxGEN-geo classifier), including comparative evaluation against an alternative classifier, namely Bernoulli Naïve Bayes.

For the purposes of classifying a sample by its country of origin, we defined the following requirements for the **vivaxGEN-geo classifier**:

1. **Capable of evaluating existing SNP panels**. The rationale was to enable continued use of SNP panels which have already been established in malaria-endemic settings as establishing SNP panels is not a trivial process.

2. **Amenable to new SNP additions (extensions) to existing SNP panels**. The rationale was that existing SNP panels can be readily optimized (rather than completely replaced) with the addition

of new SNPs as they are identified, for example, as new genomic data reflecting new countries or enhancing existing sample size are identified, or as genetic shifts are observed over time.

3. **Able to analyze data inputs containing heterozygous genotype calls or genotype fails**. Polyclonal infections are common in some malaria-endemic settings, and may exhibit heterozygous genotype calls (i.e., two or more alleles at a SNP position). Particularly in infections with low parasite densities, genotype failures (i.e., missing data at a given SNP position) may also be observed.
4. **Able to provide confidence values of the prediction from input data**. The rationale was that the end user (such as a malaria control program officer) can identify when a prediction has low confidence and is therefore able to exercise caution in interpretation of the output.

We identified the Naive Bayes classifier as having the potential to address our classifier requirements after application of several modifications.

Overview of Naive Bayes classifier

Naive Bayes is a well-described classifier, whose classification rule follows the Bayes Theorem[7]:

$$max\ Pr(C|X) \sim \frac{Pr(C)\,Pr(X|C)}{Pr(X)} \tag{1}$$

In Bayesian terminology, *Pr(C|X)* is the *posterior probability* of being class *C*, *Pr(C)* is the *prior probability* of being class *C* and the *Pr(X|C)* is the *likelihood* of being class *C* given a set of data *X*, where *C* is a class and *X* is the set of data $\{x_1, x_2, \ldots, x_n\}$ representing each of *n* feature to be used for classifying the data. With genomic data, a feature will be represented by a SNP marker or position and the data is the allele in that position, so that *X* is *{allele at marker-1, allele at marker-2, …, allele marker-n}*. *Pr(X)* is the probability that the data input *X* occurs in the set, and since it will be identical regardless of the class, it can be dropped from the classification rule to yield:

$$max\ Pr(C|X) \sim Pr(C)\,Pr(X|C) \tag{2}$$

The prior probability *Pr(C)* is usually calculated from the proportion of samples of *class C* over total training data, which presumptively represent the proportion of the circulating samples of *class C* over total population. However, for genomic data from various regions and studies, the proportion of samples in the training dataset does not necessarily represent the proportion of the circulating samples over total population, but reflects on the quality of processed samples, the number of studies performed on a region, and many other factors. Hence, for the purpose of classifying by country of origin, the prior probability will be set to uniform distribution (uniform values for all countries) so it can be dropped from the equation and that the classification rule only depends on the likelihood part of the formula, which effectively makes the classification rule equivalent to maximum likelihood:

$$max\ Pr(C|X) \sim Pr(X|C) = \prod_{i}^{n} Pr(x_i|C) \tag{3}$$

Naive Bayes can handle data with missingness by omitting the features having the missing data from the corresponding sample. For example, if the 3rd feature out of 5 features has missingness, then the calculation for *Pr(X|C)* of this sample would be:

$$Pr(X|C) = Pr(x_1|C)\,Pr(x_2|C)\,Pr(x_4|C)\,Pr(x_5|C) \tag{4}$$

Assumption on Naive Bayes is that each feature is independent, hence the naiveness of this algorithm. In reality, each feature is not really independent between each other. In practice, it has been shown that even though this assumption is frequently violated, Naive Bayes still delivers good prediction performance[8].

Classifying biallelic SNP data with Bernoulli Naive Bayes (BNB)

Binomial-based Naive Bayes is a Naive Bayes whose likelihood is a binomial probability distribution. The binomial distribution with parameters *n* and *p* is the discrete probability distribution of the number of getting one of the two possible outcomes (such as the head or tail of a coin) in a sequence of *n* independent experiments, with *p* as the probability of getting one of the outcomes. **Bernoulli Naive Bayes (BNB)** is a well-known Naive Bayes classifier whose likelihood formula is the binomial *n*=1 distribution known as Bernoulli distribution, and is being used when the input data comprises values of 0 or 1.

Biallelic SNP data can be represented by 2 states, reference allele and alternate allele, so it is suitable to be used with binomial-based Naive Bayes. A haploid sample, which only has a single set of chromosomes, can have either 0 alternate allele (when the allele is reference allele) or 1 alternate allele in a single SNP position. Obtaining the allele of a haploid sample from a SNP position can be thought of as a single independent experiment, which corresponds to a binomial probability distribution with N=1 or Bernoulli distribution. Hence, haploid samples can be classified with BNB.

The classification rule for BNB used for biallelic haploid SNP data after omitting the prior probability is:

$$max\ Pr(C|X) \sim Pr(X|C) = \prod_{i}^{n} Pr(x_i|C) = \prod_{i}^{n} p_i^{x_i}(1-p_i)^{(1-x_i)} \quad (5)$$

where p_i is the proportion of alternate allele at position *i* for country *C* and x_i is the number of occurrences of alternate allele at position *i* in the input data, with maximum $x_i = 1$.

Classifying with Biallele Likelihood (BALK) classifier

One of the requirements for the classifier in this analysis is the ability to analyze heterozygous alleles. Heterozygous alleles can arise in samples having polyclonal infection of 2 or more strains. If each sample can be represented as diploid with 2 sets of chromosomes regardless of its polyclonality, then obtaining the allele from a SNP position out of those 2 sets of chromosomes can be thought as 2 consecutive independent experiments, and hence a binomial distribution with N=2 can be used for the outcome probability of this data. The SNP data then can be stated as the number of occurrences of alternate allele in each respective position with homozygous reference allele stated as 0 (0 alternate allele, 2 reference alleles), heterozygous as 1 (1 alternate allele, 1 reference allele), and homozygous alternate allele as 2 (2 alternate alleles, 0 reference allele).

By using a binomial distribution with N=2, the classification rule for biallelic SNP allowing heterozygous call therefore becomes:

$$max\ Pr(C|X) \sim \prod_{i}^{n} \binom{n}{x} p_i^{x_i}(1-p_i)^{(2-x_i)} \quad (6)$$

Omitting the binomial coefficient, as the number would be identical across all classes, then:

$$max\ Pr(C|X) \sim \prod_{i}^{n} p_i^{x_i}(1-p_i)^{(2-x_i)} \quad (7)$$

where p_i is the proportion of alternate allele at position *i* for country *C* and x_i is the number of occurrences of alternate allele in *i* position, with max $x_i = 2$. We denote this derived classifier as **Bi-Allele Likelihood classifier (BALK)** to emphasize that it is suitable for bi-allelic SNP data from diploid samples (or any samples that can be treated as diploid for analytic purposes such as *P. vivax* clinical samples) and that the classifier only depends on the likelihood part of the Naive Bayes equation. In essence, BALK is a *N*=2 Binomial Naive Bayes whose prior probability is dropped from the classification rule.

Evaluating the performance of BNB and BALK classifiers

To illustrate the performance of BNB and BALK, a **illustrative 4-population dataset** consisting of samples from Colombia, Ethiopia, Indonesia and Thailand, each with 30 randomly-selected samples and

representing different continents, was constructed from the high quality data training set (Dataset 3), with heterozygous SNPs set to their major alleles for BNB. We used BR38 SNP panel which, although not designed for country classification, was good enough for the purpose of comparing BNB and BALK.

The classification performance was measured using the MCC (Matthew Correlation Coefficient) scores for each country. The MCC provides a measure of the quality of the classifications, ranging from -1 (total disagreement) to 1 (perfect prediction). In addition to country-level MCC scores, pooled (cross-country) median and minimum MCC scores were also determined.

For comparative analysis of classification performance, we employed a **stratified k-fold cross-validation** method. K-fold cross-validation is a resampling procedure used to evaluate the performance and model over-fitting of an untrained classifier on a limited dataset, where model here refers to the feature set, classifier and any parameter needed for the classifier. The stratified k-fold cross-validation method splits samples to *k* groups while ensuring that each group contains approximately the same proportion of samples of each target class (hence, stratified) and then use one of the group as testing set and the rest of the groups as training set iteratively and consecutively. When the number of samples for a class are less than *k*, the samples are replicated as necessary so that the sample number is equal or more than *k*. This cross-validation process can be repeated to assess the performance with different member of each group.

The following pseudocode illustrate the generic steps of repeated stratified k-fold cross-validation combined with MCC scoring above:

```
Prepare dataset X
SET random generator with a seed
REPEAT
        Split samples to k group with random stratification strategy
        FOR each group
                SET the group as testing set
                SET the remaining groups as training set
                Train classifier with training set
                Predict testing set using the trained classifier
                Calculate MCC score for each class from the prediction
                Collect all calculated MCC scores
        Calculate median MCC score from the collected MCC scores
        Calculate minimum MCC score from the collected MCC scores
UNTIL r repeats
```

The first step in the pseudocode, which is to prepare random generator with a seed, ensures that the iterative random splitting of the dataset will result in the same splits when this procedure is being used for different classifier or different feature sets and the random seed is identical. By following the above procedure, for each repeat, there will be a **median MCC score, a minimum MCC score and *k* MCC scores for each class**. These MCC scores then can be visualized using proper plot types such as boxplots.

To compare the performance of BNB and BALK, a stratified 5-fold cross-validation with the 4-population dataset and BR38 panel was performed for 100 repeats on both BNB and BALK. MCC scores for each population were plotted in the following figure:

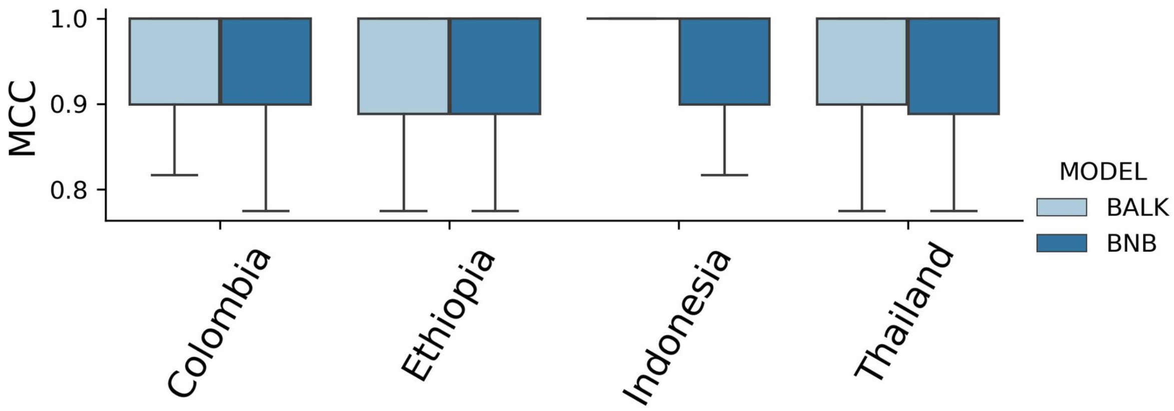

The model in the figure denoted the corresponding trained classifiers against BR38 using the 4-population dataset. The MCC scores indicated that BALK performed either equivalently or better than BNB for certain populations, such as the Indonesian samples. The Indonesian samples have been previously shown to have high levels of heterozygosity (i.e., polyclonal infections), with the models demonstrating that BALK could effectively capture the complexity in these populations.

SNP Selection

An overview of the SNP selection steps is outlined in section *b. SNP Selection* of the flowchart presented in **Figure 1**. The dataset was subjected to the following SNP selection approaches: DecisionTree, and both HFST-0.90 and HFST-0.95 (HFST with Fst threshold of 0.9 and 0.95 respectively), described in detail in the following section. For each approach, we used Dataset 3 for both training and testing set and then collected the top-25 SNP sets, ranked based on the average of their minimum MCC and median MCC scores. The top-25 SNP sets were then subjected to the repeated stratified 10-fold cross-validation, and re-ranked based on their average minimum MCC and median MCC scores to obtain the best-performing SNP set for each approach.

SNP selection using DecisionTree

DecisionTree (DT) works by selecting the best features given the data set and then using the selected features to perform the classification simultaneously. The features selected by DT can also be used by other algorithms, such as Naive Bayes, to be used for training and inferring the class of each sample. It should be noted that selected features which give the best prediction scores (MCC) with DT do not necessarily give the best prediction score with Naive Bayes. Likewise, selected features which give non-optimal prediction scores with DT do not necessarily mean that those features will give mediocre prediction scores with Naive Bayes. Hence, iterative or repetitive DT should be performed to obtain sets of selected features which then can be validated and scored using Naive Bayes classifier, or specifically BALK for this study.

For this study, the DecisionTree (DT) was not chosen as a classifier, only as a SNP selector. The rationale is that DT does not work effectively with missing data without introducing bias and has difficulties with heterozygous SNP data. To use DT, all heterozygous SNPs must be converted to their major alleles, which would reduce the actual diversity of the data and might also be problematic when one cannot confidently call the major allele from the experimental data. Working with missing data with DT means that either (i) the features with missing data or the samples with missing data are removed from the overall analysis, (ii) the missing data is treated as one of the possible outcomes, or (iii) the missing data is imputed or substituted using other methods, most likely based on probabilistic methods. All these methods would incur added biases to the original data. On the other hand, Naive Bayes classifiers such as BALK are already based on probabilistic methods and features with missing data can be omitted at individual sample to not incurring other significance biases.

The following pseudocode illustrates the generic steps in selecting the features (or SNPs) using DecisionTree and scoring the features using BALK:

```
Prepare training data X
Convert all heterozygous calls to major allele as data Xm
REPEAT
       Train a DecisionTree with data Xm
       Get feature set (SNPs) from the trained DecisionTree
       Derive new dataset X' from data X which only contains the feature set (SNPs)
       Train a BALK classifier with data X'
       Predict data X' using the trained BALK classfier
       Calculate MCC scores for each class from the above prediction
       Calculate median MCC and min MCC from the above MCC scores
       Collect the MCC scores, median MCC, min MCC and feature set
UNTIL r repeats
```

In this study, we subjected Dataset 3 to the above procedure with 500 repeats using a custom Python script which utilized DecisionTree implementation from **sklearn** library version 0.24.2, employing an optimized version of the CART (Classification And Regression Tree) algorithm and Gini coefficient. To avoid over-fitting, a minimum of 3 samples was required for a leaf node. After collecting 500 SNP sets and their corresponding MCC scores, the SNP sets were ranked based on the average of their minimum MCC and median MCC scores. The top-25 SNP sets were then subjected to stratified 10-fold cross-validation steps to select the best SNP set by ensuring that the selected SNP set would not be over-fitting to the full dataset. We denoted the best SNP set from this approach as **GEO55** since the set consisted of 55 SNPs.

SNP selection by F_{ST}

Naive Bayes works best with features that can distinguish between classes. Hence, SNPs that have unique distinct alleles which can differentiate a population against the other population are the best candidates to be used with Naive Bayes, regardless of the biological properties of the SNPs. Such SNPs are usually very low diversity or even monomorphic within the population, and hence would not be suitable for fingerprinting purposes. One of the metrics in genomic data that can be used to infer unique alleles for differentiating sub populations is F_{ST}.

Whole F_{ST} approach

A straightforward method to employ F_{ST} is by selecting SNPs with highest F_{ST} for each population within the total dataset, or whole F_{ST} of each population. To illustrate, with the previously described 4-population dataset, the SNP selection would follow the following pseudocode:

```
Select 1 SNP with highest Fst of Colombia vs rest of sample
Select 1 SNP with highest Fst of Ethiopia vs rest of sample
Select 1 SNP with highest Fst of Indonesia vs rest of sample
Select 1 SNP with highest Fst of Thailand vs rest of sample
```

The above procedure would yield 4 SNPs to be used as the feature set for training and prediction. If desired, 2 SNPs with highest F_{ST} for each step can be selected to ensure the redundancy of the SNP set in case there is any missing data in obtaining the SNPs from the experimental procedure.

The problem with this procedure is that the SNP with highest F_{ST} from a population against the rest of populations might not be able to distinguish that particular population from a closely related population. For example, the chosen SNP for Indonesia and the chosen SNP for Thailand might not be able to distinguish between Indonesia and Thailand population since the chosen SNPs would most likely lean toward differentiating Indonesia (or Thailand) against the combined Colombia and Ethiopia.

Pairwise F_{ST} approach

Another method to select SNPs by F_{ST} is to get the highest F_{ST} per pairwise population. With this method, all possible SNPs that can be used to distinguish each population from every other population can be selected. The following pseudocode illustrates the procedure to select the SNPs:

```
Select 1 SNP with highest Fst of Colombia vs Ethiopia
Select 1 SNP with highest Fst of Colombia vs Indonesia
Select 1 SNP with highest Fst of Colombia vs Thailand
Select 1 SNP with highest Fst of Ethiopia vs Indonesia
Select 1 SNP with highest Fst of Ethiopia vs Thailand
Select 1 SNP with highest Fst of Indonesia vs Thailand
```

The above procedure would yield 6 SNPs to be used as the feature set.

The pairwise F_{ST} approach ensures that there will be at least a SNP that can differentiate a population with closely related populations. However, as the number of populations (countries) increases, the number of selected SNPs increases. For example, if 1 SNP were selected for each pairwise population comparison, then for 15 populations, there would be 105 pairwise comparisons yielding 105 selected SNPs. This defeats the objective to obtain the most parsimonious number of SNPs as required for this study.

Hierarchical F_{ST} (HFST) approach

A more effective method can be devised by combining the whole F_{ST} and pairwise F_{ST} approaches. The Hierarchical F_{ST} (HFST) works by selecting SNPs using the relatedness between each population, which can be inferred by other methods such as using a neighbor-joining population tree generated by Nei's population distance. As an example, assuming that isolates from Indonesia and Thailand were closely related, and that Colombia and Ethiopia were relatively close to each other compared to Indonesia/Thailand isolates, a rooted neighbor-joining population tree constructed from the 4-populations dataset would be:

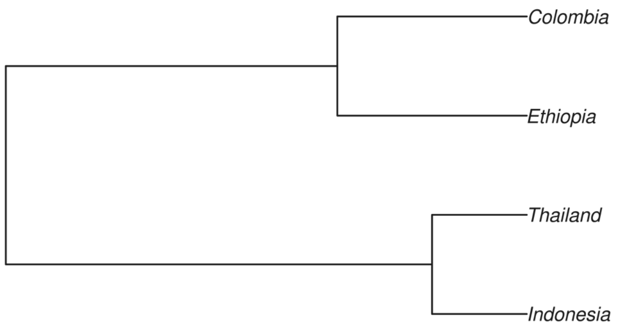

Based on the guide tree above, the following pseudocode illustrates the procedure of Hierarchical F_{ST}:

```
Select 1 SNP with highest Fst of (Indonesia ∪ Thailand) vs (Colombia ∪ Ethiopia)
Select 1 SNP with highest Fst of Colombia vs Ethiopia
Select 1 SNP with highest Fst of Indonesia vs Thailand
```

The above procedure would select 3 SNPs to be used as the feature set.

Selecting SNPs with highest F_{ST} might not be the best option as feature sets when combined with the rest of selected SNPs because SNP with the highest F_{ST} for a particular group differentiation, such as population A against B, when combined as a panel, might give detrimental effect to other group differentiation, such as population C against D. To minimize this problem, random selection and iteration is employed to find the best combination. A threshold can be used in HFST and the featured SNPs can be selected randomly based on this threshold value. For example, with a threshold of $F_{ST} > 0.95$, all SNPs within this threshold can then be chosen randomly. When none of SNPs are within this threshold value in any of the F_{ST} selection steps, a DecisionTree (DT) step can be introduced for this particular step to differentiate between the 2 nodes of the respective branch and the featured SNPs are obtained from the DT feature set. F_{ST} threshold setting can be used to influence the number of selected SNPs since increasing the F_{ST} threshold will increase the likelihood of using DT, and as DT might emit more than 1 SNP for its feature set, the number of selected SNPs will increase as well. In addition, by setting the F_{ST} threshold > 1.0, the HFST method is essentially became a guided DecisionTree.

To illustrate the above concept with the 4-population set, assuming that that there were no SNPs with F_{ST} > certain threshold both for comparison of group (Indonesia vs Thailand) against (Colombia vs Ethiopia) and for Indonesia against Thailand, the procedure would be:

```
Select SNPs using DT for (Indonesia ∪ Thailand) against (Colombia ∪ Ethiopia)
Select 1 SNP with highest Fst of Colombia vs Ethiopia
Select SNPs using DT for Indonesia vs Thailand
```

The following pseudocode illustrates the generalization of the HFST approach:

```
Prepare dataset X
Convert all heterozygous calls to major allele as dataset Xm
Read guide tree
REPEAT
        FOR each branch in guide tree from root
                Calculate Fst of all SNPs between the 2 node populations with X
                IF there are SNPs with Fst > threshold
                        Select and collect the SNPs with Fst > threshold randomly
                ELSE
                        Perform DT to classify the 2 node populations with Xm
                        Collect the feature SNPs from DT
        Derive new dataset X' from X which only contain the collected SNPs
        Train a BALK classifier with data X'
        Predict data X' using the trained BALK classifier
        Calculate MCC scores for each class from the above prediction
        Calculate median MCC and min MCC from the above MCC scores
        Collect the MCC scores, median MCC, min MCC and the collected SNPs
UNTIL r repeats
```

By combining random selection and DT method, a stochastic process is introduced to HFST and hence by repeating the HFST process, SNP sets (or feature sets) can be collected and ranked based on their MCC scores.

To create a guide tree in this study, we calculated the genetic distance matrix of all samples in Dataset 3 using all SNPs. Based on the genetic distance matrix, we computed the population genetic distance matrix using Nei's net average number of differences between populations (D_A)[9]. D_A between population 1 and 2 is defined as:

$$D_A = D_{12} - \frac{D_1 + D_2}{2} \tag{8}$$

with D as the raw number of nucleotide differences between populations, defined as:

$$D = \hat{\pi}_{12} = \sum_{i=1}^{k} \sum_{j=1}^{l} x_{1i} x_{2j} \delta_{ij} \tag{9}$$

where k and l are the number of distinct haplotypes in population 1 and 2 respectively, x_{1i} is the frequency of the i-th haplotype in population 1, x_{2j} is the frequency of the j-th haplotype in population 2, and δ_{ij} is the genetic distance (or proportion of number of allele differences) between haplotype i-th and haplotype j-th.

Alternatively, D can also be computed as:

$$D = \hat{\pi}_{12} = \frac{1}{m \times n} \sum_{i=1}^{m} \sum_{j=1}^{n} d_{ij} \tag{10}$$

where m and n are the number of samples in population 1 and 2 respectively, and d_{ij} is the genetic distance between sample i in population 1 and sample j in population 2.

Based on the Nei's net average population genetic distance matrix, a neighbor-joining tree was reconstructed using Ape, and the resulted tree was rooted as shown in Supplemental Figure 4. This rooted tree was used as the guide tree for HFST method with arbitrarily chosen F_{ST} threshold of 0.90 and 0.95, denoted as HFST-0.90 and HFST-0.95 respectively. We subjected Dataset 3 to both HFST-0.90 and HFST-0.95

for 500 repeats each using custom Python script which utilized DecisionTree as implemented in sklearn 0.24.2, to obtain 500 SNP sets for each threshold. To avoid overfitting, a maximum tree depth of 2 was set for each of the necessary DT step. The 500 SNP sets of each threshold were ranked based on the average of minimum and median MCC scores. The top-25 SNP sets of each threshold were then subjected to stratified 10-fold cross-validation steps to select the best SNP set by the average of minimum and median MCC scores, resulting in **GEO33** (33 SNPs) and **GEO50** (50 SNPs) for HFST-0.90 and HFST-0.95, respectively.

Evaluation and Assessment

An overview of the steps involved in the comparative evaluation of the SNP panels is outlined in section *c. Assessment and Validation* of the flowchart presented in **Figure 1**. Briefly, **Dataset 4** (which contained the same samples as Dataset 3 but only comprised SNPs from BR38 and selected SNP sets) was used for comparative assessment using stratified k-fold cross-validation across all SNP sets and their combination with BR38 (i.e., BR38, GEO33, GEO50, GEO55, GEO33+BR38, GEO50+BR38 and GEO55+BR38). The data missingness assessment was also performed using Dataset 4. Independent validation was undertaken using the **Independent Validation Dataset**. All metrics were based on MCC scores.

Comparative assessment of candidate SNP panels

To compare the BR38 SNP panel to the three new candidate SNP panels identified by pure DT, HFST-0.90, and HFST-0.95 approaches, a 500 repeat, stratified 10-fold cross-validation was undertaken on each SNP panel and its combination with BR38 (i.e., BR38, GEO33, GEO50, GEO55, GEO33+BR38, GEO50+BR38 and GEO55+BR38) using Dataset 3. The MCC scores were reported as **Figure 2** and **Table 1**.

Assessment of the durabililty of candidate SNP panels

To assess the durability of prediction performance of the candidate SNP panels with different levels of missing data (analogous to genotyping failures), repetitive predictions were run after removing genotype data randomly. The BALK classifier was trained with all samples from Dataset 3 against the candidate SNP panels. To prepare the testing sample set, 25 samples were sampled randomly with replacement for each country from Dataset 3 and genotype calls were randomly removed from the sampled data in 10%, 20% and 30% proportions. The random samples with random missing data were then predicted by the trained classifier. This process was run in 250 repeats and the MCC score of the prediction for each country was reported in **Figure 3** and **Supplementary Table 6**.

The following pseudocode illustrates the detailed steps of the above process:

```
Prepare dataset X
FOR each snp set
        SET random_generator_1 with seed
        Create dataset X’ which only contain snps from current snp set
        Train BALK classifier with dataset X’ against current snp set
        REPEAT
                FOR each population in dataset X’
                        Use random_generator_1 to random sampling with replacement
                        Collect the 25 samples to dataset Z
                FOR proportion in 0.1, 0.2, 0.3
                        Copy dataset Z to Z’
                        For each sample in dataset Z’
                                Remove random allel with proportion
                        Predict each sample in dataset Z’ using trained BALK
                        Calculate MCC scores for each population
                        Collect all MCC scores, proportion and snp set
        UNTIL 250 repeats
Plot MCC scores per population with snp set and missingness proportion
```

Validation using independent dataset

To evaluate the performance of the candidate SNP panels with new sample sets (as opposed to using the re-sampling technique of the cross-validation strategy), the BALK classifier was trained with all samples from Dataset 3 against all candidate SNP panels, including their combination with BR38 (i.e., GEO33, GEO50, GEO55, GEO33+BR38, GEO50+BR38 and GEO55+BR38). Each sample from the Independent Validation Dataset was then predicted by the trained BALK classifiers for each panel set. MCC scores for each country were reported as **Table 2** and prediction results were plotted as heatmaps in **Figure 4**.

Web-based Data Analysis Platform for End-Users

To establish accessible informatics tools for end users, an online platform was created incorporating data classification tools for determining the most likely country of origin of a sample using genetic data derived from different barcodes. Existing source code, developed for a microsatellite-based *P. vivax* data sharing and analysis platform [10], was modified to create a new web-based platform (**vivaxGEN-geo**), to analyze SNP data generated at the geographic barcode. This approach was chosen owing to the ability to i) incorporate manual SNP sets allowing incremental improvements of the barcode in future, ii) evaluate barcodes with incomplete data owing to genotyping failures, and iii) evaluate heterozygous genotype calls, which reflect polyclonal infections. For optimal accuracy, the BALK classifier provided on the online platform has been trained with 941 samples, comprising Dataset 2 (N=799) plus the Independent Validation Dataset (N=142). The classifier tool reports the three highest likelihoods for country of origin and their associated probabilities. To provide better estimates of probability from Naïve Bayes classifier, the probabilities were computed using isotonic method as implemented in **CalibratedClassfierCV** of sklearn library, with stratified 4-fold cross-validation for the calibration dataset[11]. The web platform is able to receive the input data as string-based barcode representation, column-based tab-delimited text files, and VCF files.

Implementation and Web Service Availability

All custom scripts were implemented in Python, except when noted otherwise, and were run under Python 3.9. All custom scripts to process VCF files and other data are available at https://github.com/trmznt/pys. The Bernoulli Naive Bayes (**NaNBernoulliNB**) and Biallele Likelihood (**BialleleLK**) classifiers, both with missing data capability, is implemented in Python and available at https://github.com/trmznt/lkc-bisnp. Both classifiers were written in sklearn-type style and could be used directly in a similar way as the rest of classifiers implemented in sklearn. All analysis was performed with sklearn version 0.24.2 under Python 3.9. The version on website had been updated using Python 3.10 and sklearn version 1.1.1.

Supplementary Tables

Supplementary Table 1. Summary of number of samples per country

Country	Dataset 0 (N)	Dataset 1 (N)	DataSet 2 (N)	Dataset 3 & 4 (N)	IVD (N)
Afghanistan	250	244	48	48	-
Bangladesh	28	28	8	8	-
Bhutan	9	9	4	4	-
Brazil	25	25	10 (4)	10	7
Cambodia	123	115	110	110	65
China	5	5	5	5	-
Colombia	107	88	50	50	1
Ethiopia	43	42	33	33	18
India	14	14	7	7	-
Indonesia	280	250	211	211	-
Iran	15	15	9	9	-
Madagascar	4	4	4	4	-
Malaysia	107	62	40	38	-
Mexico	20	18	18	15	-
Myanmar	9	9	8	8	-
Papua New Guinea	30	29	19	18	-
Peru	47	46	41	37	14
Philippines	6*	6	4	4	-
Sudan	13	13	4	4	-
Thailand	125	123	122	119	12
Vietnam	88	82	71	57	25
Total	1348	1227	826	799	142

The initial dataset (Dataset 0) consisted of patient samples in the MalariaGEN Plasmodium Vivax Community Project release 4 (Pv4) derived from previous publications (i.e., prior to the Pv4 open access release) as well as unpublished samples from Menzies partner sites, and 6 Philippines samples from GSK dataset, excluding the recurrent infections. The independent patient samples (Dataset 1) comprised all infections from the initial Dataset 0 after filtering out isolates from pairs with nucleotide genetic distance less than 0.001 (0.1%). The high-quality dataset (Dataset 2) consisted of the 826 biologically independent patient samples with complete data at the 229k high-quality SNPs. The training dataset (Dataset 3) consisted of high-quality samples after cleaning-up using predictions based on BALK classifier using all 299k SNPs and manual confirmation using a neighbor-joining tree. The GSK validation samples (IVD – Independent Validation Dataset) comprised independent samples that were not included in the training dataset.

Supplementary Table 2. Details of the GEO33 barcode markers

Chromosome	Position	Reference allele	Alternative allele	Gene	Amino acid change
PvP01_03_v1	220999	C	G	*PVP01_0304100 (DPAP3)*	S470T
PvP01_05_v1	693449	T	G	*PVP01_0516600*	S714A
PvP01_05_v1	1079461	G	T	-	-
PvP01_05_v1	1285366	T	C	*PVP01_0530500*	-
PvP01_06_v1	646186	T	C	*PVP01_0615300 (CLAMP)*	K293E
PvP01_07_v1	261070	A	G	*PVP01_0704300 (SEC27)*	L125
PvP01_07_v1	595236	G	A	-	-
PvP01_08_v1	442363	G	T	*PVP01_0809900*	R1312
PvP01_08_v1	879062	T	A	-	-
PvP01_08_v1	1420994	T	A	*PVP01_0833200*	G106
PvP01_09_v1	303291	C	T	*PVP01_0905000 (SNF2L)*	G876E
PvP01_09_v1	448825	C	T	*PVP01_0908400*	G533E
PvP01_09_v1	1101235	G	A	*PVP01_0924700*	H1141
PvP01_09_v1	1884013	T	G	*PVP01_0942600 (IMC1b)*	E141D
PvP01_10_v1	480601	G	A	*PVP01_1010900 (MDR1)*	L845F
PvP01_10_v1	483568	G	A	-	-
PvP01_10_v1	1131031	G	A	*PVP01_1025900 (SET9)*	I908
PvP01_11_v1	384043	C	T	*PVP01_1109600*	E958K
PvP01_11_v1	503992	A	T	*PVP01_1112200*	F3054I
PvP01_11_v1	1137410	C	T	-	-
PvP01_11_v1	1226906	G	T	*PVP01_1128100 (ALV7)*	F583L
PvP01_11_v1	1448769	A	G	*PVP01_1133700*	L1638
PvP01_11_v1	1868012	C	A	*PVP01_1144100*	R314
PvP01_12_v1	1116377	C	T	*PVP01_1227900*	S1142
PvP01_12_v1	1400307	A	T	*PVP01_1235200*	N1141K
PvP01_12_v1	1463080	G	A	*PVP01_1236900*	A209V
PvP01_12_v1	1988355	C	T	*PVP01_1249000*	K2631
PvP01_13_v1	66121	T	A	*PVP01_1301600*	I1200
PvP01_14_v1	344881	T	G	*PVP01_1407300 (RPT4)*	V350
PvP01_14_v1	1160762	C	T	*PVP01_1427200*	P85L
PvP01_14_v1	1229487	G	T	*PVP01_1428700*	S1136I
PvP01_14_v1	1258544	C	T	-	-
PvP01_14_v1	1266326	A	T	-	-

Supplementary Table 3. Details of the GEO50 barcode markers

Chromosome	Position	Reference allele	Alternative allele	Gene	Amino acid change
PvP01_01_v1	746509	G	A	*PVP01_0116500 (g-tub)*	D130
PvP01_02_v1	491114	A	G	-	-
PvP01_03_v1	764851	C	T	*PVP01_0318000*	A698T
PvP01_04_v1	400824	G	A	*PVP01_0409900 (ACS9)*	R413
PvP01_05_v1	693449	T	G	*PVP01_0516600*	S714A
PvP01_05_v1	1045059	C	T	-	-
PvP01_05_v1	1049754	G	T	*PVP01_0525800 (HAT1)*	S1071
PvP01_05_v1	1052474	G	T	-	-
PvP01_05_v1	1079461	G	T	-	-
PvP01_05_v1	1286077	G	T	-	-
PvP01_06_v1	645726	A	G	*PVP01_0615300 (CLAMP)*	F446S
PvP01_06_v1	757119	G	A	*PVP01_0618300 (ETRAMP)*	A449T
PvP01_06_v1	882660	C	T	*PVP01_0621600*	F512
PvP01_07_v1	595236	G	A	-	-
PvP01_08_v1	442363	G	T	*PVP01_0809900*	R1312
PvP01_08_v1	555644	T	C	*PVP01_0813100*	C45
PvP01_08_v1	1420994	T	A	*PVP01_0833200*	G106
PvP01_09_v1	303291	C	T	*PVP01_0905000 (SNF2L)*	G876E
PvP01_09_v1	644970	G	A	*PVP01_0913800*	S5337N
PvP01_09_v1	735276	G	T	*PVP01_0916300 (ApiAP2)*	-
PvP01_09_v1	924204	C	T	*PVP01_0921200*	S818N
PvP01_09_v1	1169264	C	T	*PVP01_0926500*	V439I
PvP01_09_v1	1884013	T	G	*PVP01_0942600 (IMC1b)*	E141D
PvP01_10_v1	165046	T	G	*PVP01_1003000*	T74
PvP01_10_v1	256817	C	A	-	-
PvP01_10_v1	480601	G	A	*PVP01_1010900 (MDR1)*	L845F
PvP01_10_v1	671286	G	A	*PVP01_1014900 (SLBP)*	T220I
PvP01_11_v1	108593	A	G	*PVP01_1102600*	V2443A
PvP01_11_v1	371608	A	C	-	-
PvP01_11_v1	550783	C	G	-	-
PvP01_11_v1	1345904	G	C	*PVP01_1131500*	N706K
PvP01_11_v1	1364199	A	C	*PVP01_1131900*	N362K
PvP01_12_v1	1695950	C	T	*PVP01_1241500*	V579M
PvP01_12_v1	2324641	C	T	-	-
PvP01_12_v1	2353408	C	A	-	-
PvP01_13_v1	699780	A	G	*PVP01_1315100*	I208V
PvP01_13_v1	1036146	C	T	-	-
PvP01_13_v1	1209242	G	A	*PVP01_1328500 (GlyRS)*	I228
PvP01_13_v1	1661666	A	C	*PVP01_1338500 (RAP1)*	Y234D
PvP01_14_v1	138349	A	C	*PVP01_1402900*	T134
PvP01_14_v1	763508	C	A	-	-
PvP01_14_v1	1229487	G	T	*PVP01_1428700*	S1136I
PvP01_14_v1	1270401	G	C	*PVP01_1429500 (PPPK-DHPS)*	A553G
PvP01_14_v1	1272043	T	A	*PVP01_1429500 (PPPK-DHPS)*	-
PvP01_14_v1	1343648	T	C	*PVP01_1430700*	G5107
PvP01_14_v1	1348961	C	T	*PVP01_1430900*	E198K
PvP01_14_v1	1349891	G	A	-	-
PvP01_14_v1	2566159	C	T	-	-
PvP01_14_v1	2907128	G	T	-	-
PvP01_14_v1	2921425	C	G	*PVP01_1468700*	L181V

Supplementary Table 4. Details of the GEO55 barcode markers

Chromosome	Position	Reference allele	Alternative allele	Gene	Amino acid change
PvP01_02_v1	355597	C	T	*PVP01_0208500 (ATP6)*	D912
PvP01_02_v1	491114	A	G	-	-
PvP01_02_v1	718939	G	T	-	-
PvP01_03_v1	184372	G	A	-	-
PvP01_03_v1	384548	A	T	*PVP01_0307900 (SIAP1)*	M822L
PvP01_03_v1	764851	C	T	*PVP01_0318000*	A698T
PvP01_03_v1	823594	G	A	-	-
PvP01_03_v1	850299	C	G	*PVP01_0320400*	V3187L
PvP01_04_v1	341631	G	A	*PVP01_0408400*	C411
PvP01_04_v1	776919	G	C	*PVP01_0418800 (AROM)*	H1712Q
PvP01_05_v1	452615	G	C	*PVP01_0510300*	V88
PvP01_05_v1	1020519	C	A	-	-
PvP01_05_v1	1278457	C	T	*PVP01_0530200*	E90K
PvP01_06_v1	757119	G	A	*PVP01_0618300 (ETRAMP)*	A449T
PvP01_06_v1	923512	C	G	-	-
PvP01_07_v1	427785	G	A	*PVP01_0708400*	D28N
PvP01_07_v1	507890	T	A	*PVP01_0709900*	P582
PvP01_07_v1	1137322	C	A	-	-
PvP01_08_v1	956216	G	C	*PVP01_0821600*	G94
PvP01_08_v1	1506827	A	G	-	-
PvP01_09_v1	339310	C	G	-	-
PvP01_09_v1	644970	G	A	*PVP01_0913800*	S5337N
PvP01_09_v1	1169264	C	T	*PVP01_0926500*	V439I
PvP01_09_v1	1547999	C	T	*PVP01_0936000*	S595N
PvP01_09_v1	1884013	T	G	*PVP01_0942600 (IMC1b)*	E141D
PvP01_10_v1	184645	G	A	*PVP01_1003600*	R448C
PvP01_10_v1	256817	C	A	-	-
PvP01_10_v1	383155	G	T	*PVP01_1008600 (SMC6)*	R104
PvP01_10_v1	480601	G	A	*PVP01_1010900 (MDR1)*	L845F
PvP01_10_v1	671286	G	A	*PVP01_1014900 (SLBP)*	T220I
PvP01_11_v1	516764	G	C	*PVP01_1112300*	A1672G
PvP01_11_v1	1724604	C	T	-	-
PvP01_12_v1	338808	G	A	*PVP01_1208400*	C3664
PvP01_12_v1	377299	T	A	*PVP01_1209100 (IDH)*	L231I
PvP01_12_v1	553265	T	A	-	-
PvP01_12_v1	882252	G	A	*PVP01_1222500*	S343
PvP01_12_v1	1127030	T	G	*PVP01_1228200*	N133K
PvP01_12_v1	1512622	G	A	*PVP01_1238200*	Y704
PvP01_12_v1	1695950	C	T	*PVP01_1241500*	V579M
PvP01_12_v1	1738799	C	T	*PVP01_1242500*	S551N
PvP01_12_v1	2057371	T	G	*PVP01_1250400*	M1539L
PvP01_12_v1	2353408	C	A	-	-
PvP01_13_v1	411903	T	A	*PVP01_1309000 (ABCB7)*	I393F
PvP01_13_v1	485833	A	G	*PVP01_1310500 (MCM8)*	Y458
PvP01_13_v1	516924	C	T	*PVP01_1311100 (ATP4)*	E1158K
PvP01_13_v1	1332969	T	C	-	-
PvP01_13_v1	1340035	G	T	*PVP01_1331500 (EPAC)*	S1029R
PvP01_13_v1	1661666	A	C	*PVP01_1338500 (RAP1)*	Y234D
PvP01_14_v1	446323	T	C	*PVP01_1409500*	N256S
PvP01_14_v1	460513	A	G	-	-
PvP01_14_v1	1229487	G	T	*PVP01_1428700*	S1136I
PvP01_14_v1	1270401	G	C	*PVP01_1429500 (PPPK-DHPS)*	A553G
PvP01_14_v1	1348961	C	T	*PVP01_1430900*	E198K
PvP01_14_v1	1349891	G	A	-	-
PvP01_14_v1	2339401	C	T	-	-

Supplementary Table 5. Summary of SNPs that overlap between GEO33, GEO50 and GEO55 panels

Chromosome	Position	Reference Allele	Alternate Allele	Gene	Amino Acid Change	BR38	GEO33	GEO50	GEO55	No. of panels overlapping
PvP01_02_v1	491114	A	G			-	-	1	1	2
PvP01_03_v1	764851	C	T	*PVP01_0318000*	A698T	-	-	1	1	2
PvP01_05_v1	693449	T	G	*PVP01_0516600*	S714A	-	1	1	-	2
PvP01_05_v1	1079461	G	T			-	1	1	-	2
PvP01_06_v1	757119	G	A	*PVP01_0618300*	A449T	-	-	1	1	2
PvP01_07_v1	595236	G	A			-	1	1	-	2
PvP01_08_v1	442363	G	T	*PVP01_0809900*	R1312	-	1	1	-	2
PvP01_08_v1	1420994	T	A	*PVP01_0833200*	G106	-	1	1	-	2
PvP01_09_v1	303291	C	T	*PVP01_0905000*	G876E	-	1	1	-	2
PvP01_09_v1	644970	G	A	*PVP01_0913800*	S5337N	-	-	1	1	2
PvP01_09_v1	1169264	C	T	*PVP01_0926500*	V439I	-	-	1	1	2
PvP01_09_v1	1884013	T	G	*PVP01_0942600*	E141D	-	1	1	1	3
PvP01_10_v1	256817	C	A			-	-	1	1	2
PvP01_10_v1	480601	G	A	*PVP01_1010900*	L845F	-	1	1	1	3
PvP01_10_v1	671286	G	A	*PVP01_1014900*	T220I	-	-	1	1	2
PvP01_12_v1	1695950	C	T	*PVP01_1241500*	V579M	-	-	1	1	2
PvP01_12_v1	2353408	C	A			-	-	1	1	2
PvP01_13_v1	1661666	A	C	*PVP01_1338500*	Y234D	-	-	1	1	2
PvP01_14_v1	1229487	G	T	*PVP01_1428700*	S1136I	-	1	1	1	3
PvP01_14_v1	1270401	G	C	*PVP01_1429500*	A553G	-	-	1	1	2
PvP01_14_v1	1348961	C	T	*PVP01_1430900*	E198K	-	-	1	1	2
PvP01_14_v1	1349891	G	A			-	-	1	1	2

Supplementary Table 6. Summary of MCC scores with 0-30% missing data

	BR38				GEO33				GEO33+BR38				GEO50				GEO50+BR38				GEO55				GEO55+BR38			
**% Missing**	**0%**	**10%**	**20%**	**30%**	**0%**	**10%**	**20%**	**30%**	**0%**	**10%**	**20%**	**30%**	**0%**	**10%**	**20%**	**30%**	**0%**	**10%**	**20%**	**30%**	**0%**	**10%**	**20%**	**30%**	**0%**	**10%**	**20%**	**30%**
Afghanistan	0.84	0.8	0.75	0.72	0.7	0.66	0.6	0.56	0.93	0.93	0.92	0.9	0.8	0.8	0.77	0.72	1	0.98	0.96	0.94	0.79	0.78	0.77	0.74	0.94	0.92	0.9	0.89
Bangladesh	0.87	0.8	0.72	0.66	1	0.94	0.87	0.77	1	1	1	0.98	1	0.98	0.94	0.89	1	1	1	0.98	1	1	1	0.98	1	1	1	1
Bhutan	1	1	0.98	0.96	0.87	0.82	0.77	0.69	1	1	1	1	1	0.96	0.89	0.82	1	1	1	1	1	0.98	0.94	0.91	1	1	1	1
Brazil	0.98	0.98	0.96	0.94	0.98	0.91	0.86	0.81	1	1	1	0.98	0.79	0.87	0.87	0.82	1	1	1	0.98	1	0.96	0.91	0.87	1	1	1	0.98
Cambodia	0.52	0.49	0.47	0.43	0.7	0.68	0.65	0.61	0.85	0.84	0.8	0.76	0.85	0.83	0.8	0.77	0.89	0.86	0.84	0.82	0.96	0.9	0.86	0.82	0.94	0.91	0.88	0.85
China	0.98	0.98	0.96	0.94	1	1	1	0.98	1	1	1	1	1	1	1	0.98	1	1	1	1	1	1	1	1	1	1	1	1
Colombia	0.96	0.9	0.86	0.82	0.98	0.94	0.87	0.82	0.98	0.98	0.97	0.96	1	0.96	0.92	0.87	1	0.98	0.98	0.96	1	0.96	0.93	0.89	1	1	1	0.98
Ethiopia	0.98	0.96	0.96	0.94	1	0.92	0.83	0.77	1	1	1	1	1	0.94	0.91	0.85	1	1	1	1	0.98	0.94	0.89	0.86	1	1	1	1
India	0.82	0.82	0.79	0.74	0.94	0.85	0.79	0.74	0.91	0.94	0.94	0.92	0.84	0.89	0.87	0.85	1	0.98	0.98	0.96	0.77	0.77	0.78	0.76	0.94	0.91	0.91	0.9
Indonesia	0.79	0.73	0.67	0.63	0.94	0.86	0.76	0.68	1	0.96	0.94	0.89	1	0.98	0.94	0.88	0.98	1	0.98	0.98	0.98	0.96	0.94	0.91	0.98	0.98	0.98	0.98
Iran	1	0.98	0.98	0.96	0.94	0.89	0.8	0.72	1	1	1	1	0.89	0.86	0.82	0.77	1	1	1	0.98	1	0.93	0.89	0.84	1	1	1	0.98
Madagascar	1	1	0.98	0.96	1	0.96	0.91	0.87	1	1	1	1	1	0.98	0.94	0.91	1	1	1	1	1	0.98	0.94	0.89	1	1	1	1
Malaysia	0.87	0.82	0.8	0.77	0.96	0.89	0.83	0.77	0.94	0.94	0.94	0.92	0.94	0.94	0.94	0.91	0.94	0.96	0.94	0.94	0.98	0.96	0.96	0.94	0.96	0.96	0.96	0.96
Mexico	1	1	1	1	1	1	0.98	0.96	1	1	1	1	1	0.98	0.96	0.92	1	1	1	1	1	1	0.98	0.98	1	1	1	1
Myanmar	0.77	0.74	0.69	0.66	1	0.85	0.72	0.63	0.94	0.91	0.9	0.87	0.94	0.84	0.8	0.72	0.94	0.91	0.89	0.87	0.94	0.91	0.87	0.84	0.94	0.94	0.94	0.94
PNG	0.74	0.65	0.56	0.48	0.98	0.94	0.87	0.82	1	0.98	0.96	0.92	1	1	0.98	0.96	1	1	1	1	1	1	1	0.98	1	1	1	1
Peru	0.91	0.89	0.87	0.84	0.98	0.9	0.82	0.74	0.98	0.98	0.96	0.94	0.85	0.88	0.83	0.78	1	0.98	0.96	0.96	1	0.95	0.91	0.87	1	1	0.98	0.98
Philippines	0.68	0.68	0.59	0.52	0.68	0.71	0.62	0.56	0.98	0.98	0.96	0.92	0.94	0.94	0.92	0.89	0.96	0.96	0.96	0.96	1	0.98	0.96	0.94	0.98	1	0.98	0.98
Sudan	1	1	1	0.98	0.87	0.79	0.71	0.68	1	1	1	1	1	1	0.98	0.96	1	1	1	1	1	0.96	0.91	0.87	1	1	1	1
Thailand	0.58	0.53	0.49	0.46	0.98	0.86	0.78	0.71	0.92	0.91	0.88	0.85	0.93	0.86	0.84	0.8	0.94	0.92	0.9	0.89	0.94	0.9	0.86	0.82	0.94	0.93	0.91	0.89
Vietnam	0.57	0.54	0.47	0.43	0.68	0.62	0.56	0.5	0.86	0.83	0.79	0.75	0.83	0.81	0.77	0.72	0.88	0.84	0.81	0.78	0.94	0.9	0.86	0.8	0.94	0.91	0.88	0.85
Pooled median	0.87	0.82	0.8	0.77	0.98	0.89	0.8	0.74	1	0.98	0.96	0.94	0.94	0.94	0.91	0.85	1	1	0.98	0.98	1	0.96	0.91	0.87	1	1	1	0.98
Pooled min	0.52	0.49	0.47	0.43	0.68	0.62	0.56	0.5	0.85	0.83	0.79	0.75	0.79	0.8	0.77	0.72	0.88	0.84	0.81	0.78	0.77	0.77	0.77	0.74	0.94	0.91	0.88	0.85

Supplementary Figures

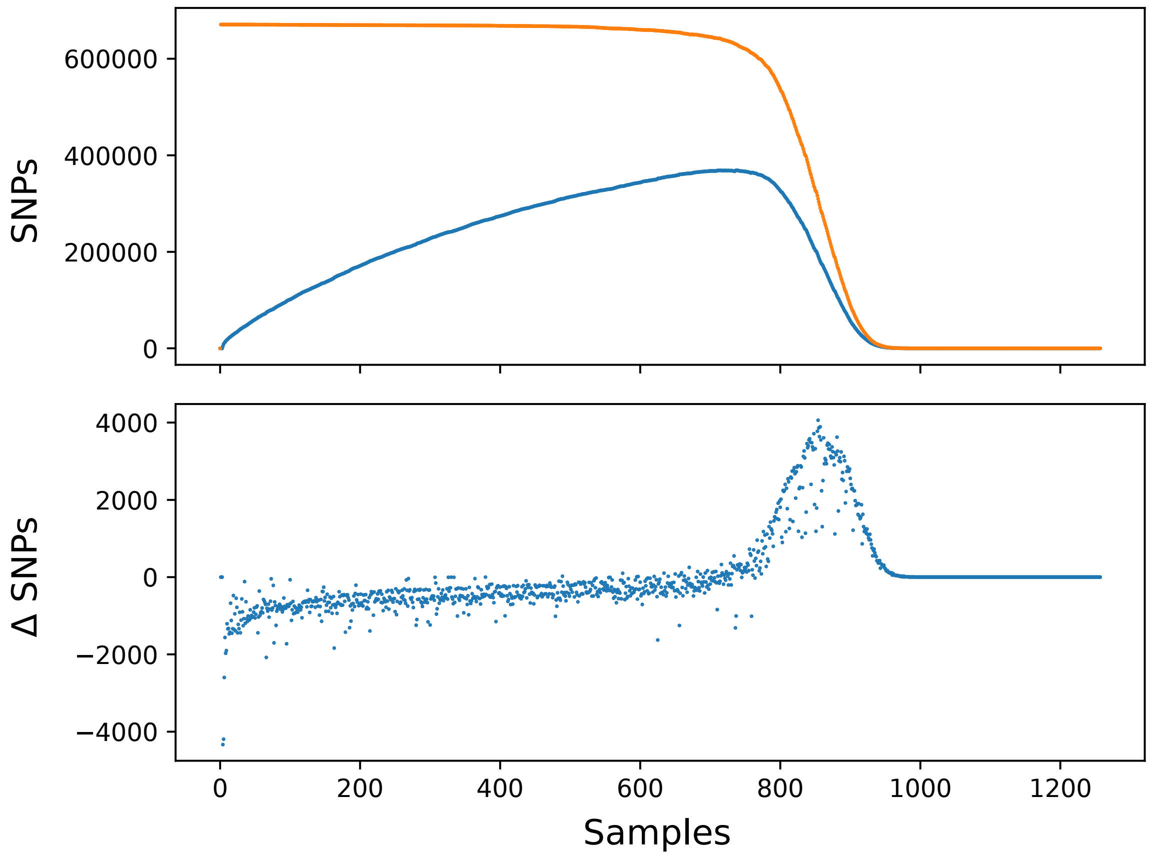

Supplementary Figure 1. Output from data quality iterative filtering process. The upper panel shows the number of SNPs (orange) without any missing data and the number of SNPs with MAC >= 2 (blue) against the number of samples in Dataset 0. The lower panel shows the changes in the number of SNPs with MAC >=2 between consecutive number of samples which peaked at 826 samples (blue dots). This data quality iterative filtering process determined the number of SNPs which had genotype calls in all samples within a sample set and for which the respective SNPs were informative, designated as SNPs with minor allele count (MAC) >= 2 by removing individual sample from highest to lowest levels of missing genotype calls iteratively from the sample set while collecting the number of remaining samples, the number of SNPs without any missing genotype calls, and the number of informative SNPs without any missing genotype calls.

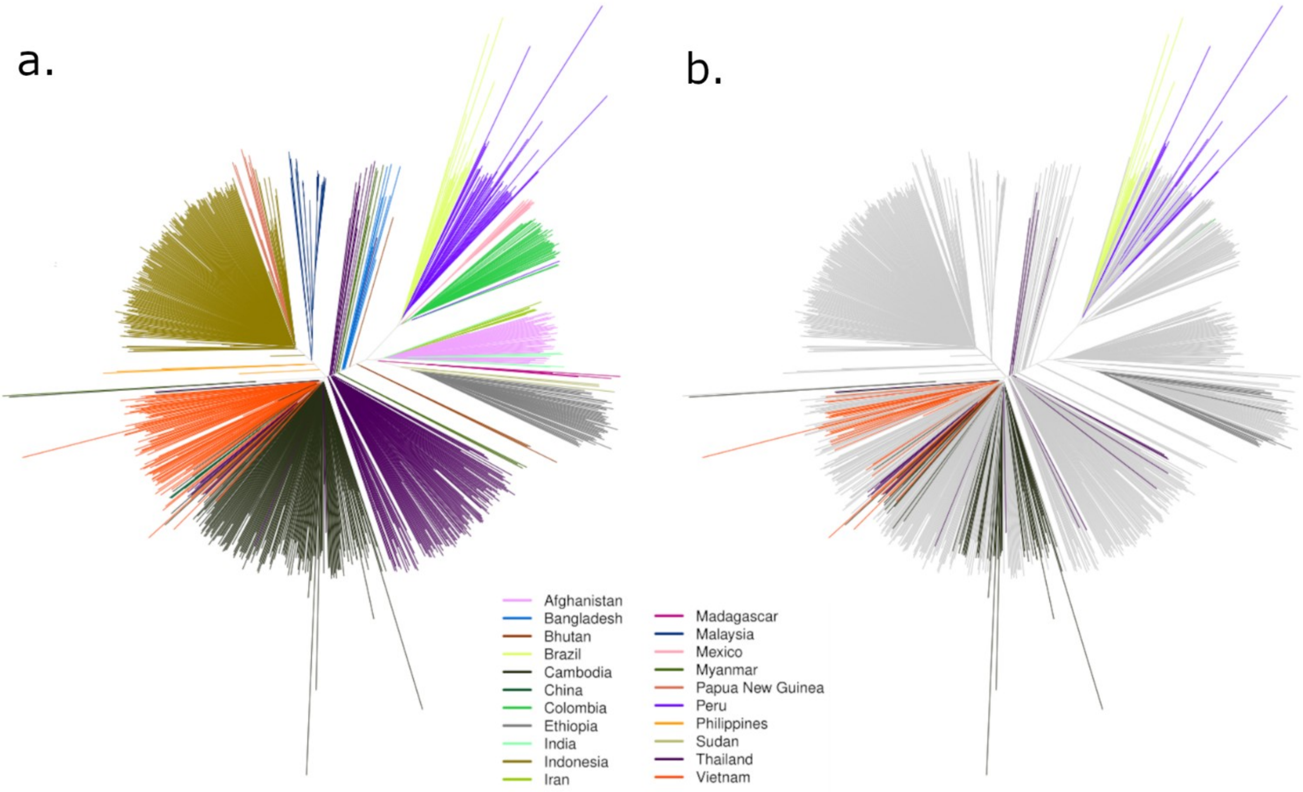

Supplementary Figure 2. Neighbor-joining trees of the combined Dataset 2 and Independent Validation Dataset. Panel a. presents the training samples consisting of 799 biologically independent samples from 21 countries (Dataset 3) in addition to the 142 independent validation samples (Independent Validation Dataset). All branches are colour-coded according to country of presentation. Panel b. presents the same tree as in panel a. but with only the independent validation samples colour-coded by country to highlight their positions (all other branches in grey). The trees were generated using the Dataset 2 SNP panel comprising 229,317 high-quality SNPs.

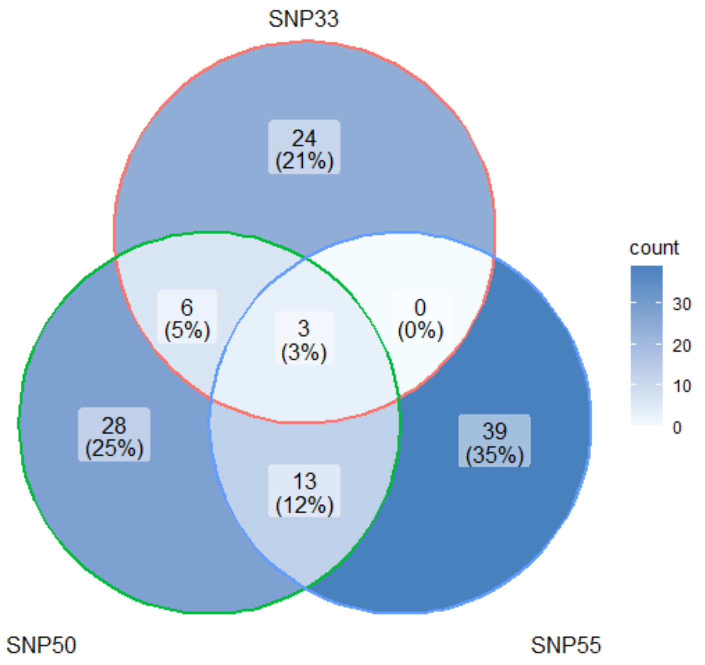

Supplementary Figure 3. SNP overlap between the SNP panels. Venn diagram illustrating the number (and percentage) of SNPs overlapping between the GEO33, GEO50 and GEO55 panels. The baseline for the percentages is the total number of SNPs across the three panels. Details of the overlapping SNPs are presented in Supplementary Table 3. For visual simplicity, the BR38 Broad barcode is not presented in the venn diagram as there was no overlap between any of the SNPs in this panel with any of the three new panels.

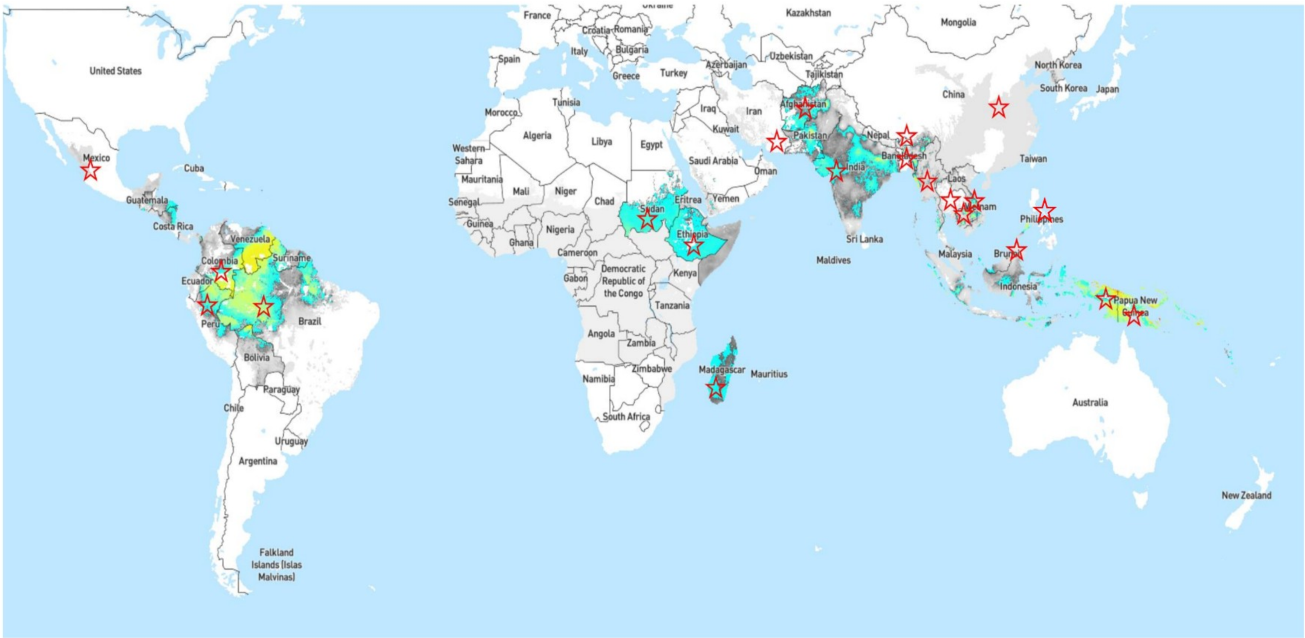

Supplementary Figure 4. ***P. vivax*** **prevalence map pinpointing the countries included in the study**. The baseline *P. vivax* prevalence map was derived from the Malaria Atlas Project (MAP) and depicts the *Plasmodium vivax* parasite rate in all ages globally (1- 99) from 2000-2017[12]. The MAP figure was modified by the addition of stars demarking the 21 countries included in Dataset 2: Mexico, Colombia, Peru, Brazil, Madagaskar, Sudan, Ethiopia, Iran, Afghanistan, India, Bhutan, Bangladesh, Myanmar, Thailand, Cambodia, Vietnam, China, Malaysia, Philippines, Indonesia, and Papua New Guinea.

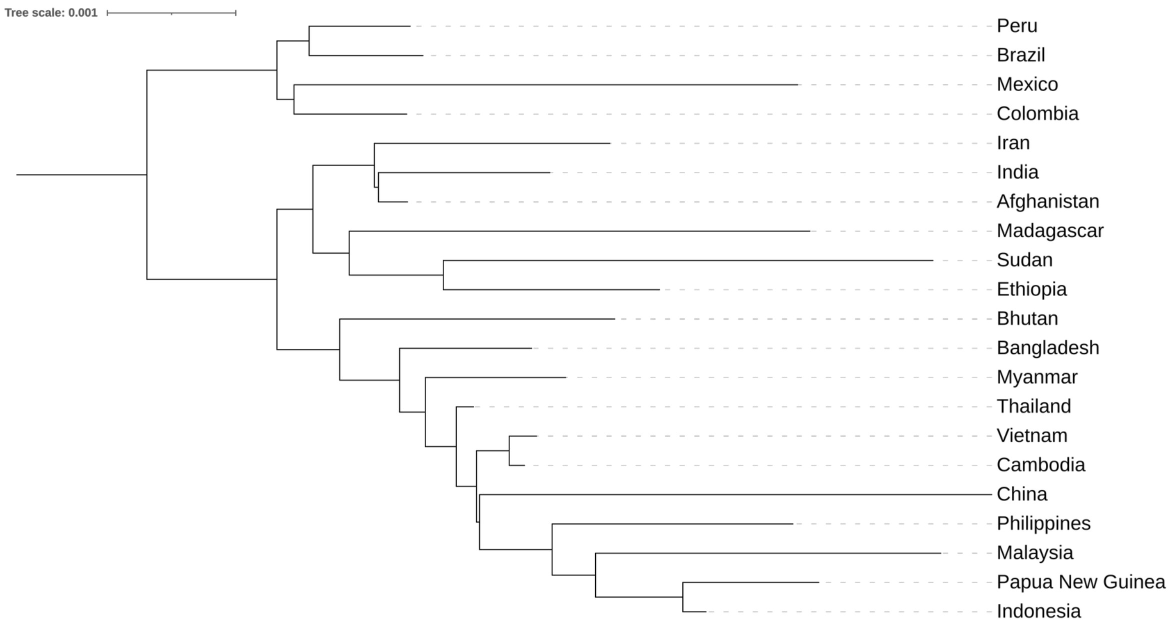

Supplementary Figure 5. Rooted neighbor-joining population tree used in HFST. The neighbor-joining tree was constructed using Ape based on a population distance matrix calculated based on Nei's net average population distance from 799 biologically independent samples. iTOL was then used to root the tree at midpoint and to plot the tree.

Supplementary References

1 MalariaGen *et al.* An open dataset of Plasmodium vivax genome variation in 1,895 worldwide samples. *Wellcome open research* **7**, 136, doi:10.12688/wellcomeopenres.17795.1 (2022).

2 Auburn, S. *et al.* A new Plasmodium vivax reference sequence with improved assembly of the subtelomeres reveals an abundance of pir genes. *Wellcome open research* **1**, 4, doi:10.12688/wellcomeopenres.9876.1 (2016).

3 Paradis, E. & Schliep, K. ape 5.0: an environment for modern phylogenetics and evolutionary analyses in R. *Bioinformatics* **35**, 526-528, doi:10.1093/bioinformatics/bty633 (2019).

4 Ba, H. *et al.* Multi-locus genotyping reveals established endemicity of a geographically distinct Plasmodium vivax population in Mauritania, West Africa. *PLoS neglected tropical diseases* **14**, e0008945, doi:10.1371/journal.pntd.0008945 (2020).

5 Baniecki, M. L. *et al.* Development of a single nucleotide polymorphism barcode to genotype Plasmodium vivax infections. *PLoS neglected tropical diseases* **9**, e0003539, doi:10.1371/journal.pntd.0003539 (2015).

6 Dewasurendra, R. L. *et al.* Use of a Plasmodium vivax genetic barcode for genomic surveillance and parasite tracking in Sri Lanka. *Malaria journal* **19**, 342, doi:10.1186/s12936-020-03386-3 (2020).

7 Mitchell, T. M. *Machine Learning*. (McGraw-Hill, 1997).

8 Lewis, D. D. Naive (Bayes) at forty: The independence assumption in information retrieval. *European Conference on Machine Learning*, doi:10.1007/BFb0026666 (1998).

9 Nei, M. & Li, W. H. Mathematical model for studying genetic variation in terms of restriction endonucleases. *Proc Natl Acad Sci U S A* **76**, 5269-5273, doi:10.1073/pnas.76.10.5269 (1979).

10 Trimarsanto, H. *et al.* VivaxGEN: An open access platform for comparative analysis of short tandem repeat genotyping data in Plasmodium vivax populations. *PLoS neglected tropical diseases* **11**, e0005465, doi:10.1371/journal.pntd.0005465 (2017).

11 Menon, A. K., Jiang, X. J., Vembu, S., Elkan, C. & Ohno-Machado, L. Predicting accurate probabilities with a ranking loss. *Proc Int Conf Mach Learn* **2012**, 703-710 (2012).

12 Battle, K. E. *et al*. Mapping the global endemicity and clinical burden of Plasmodium vivax, 2000-17: a spatial and temporal modelling study. *Lancet* Jul 27;394(10195):332–43 doi: 10.1016/S0140-6736(19)31096-7 (2019)
