# Supplementary material for: A molecular barcode and web-based data analysis tool to identify imported Plasmodium vivax malaria
Source: Commun Biol. 2022 Dec 23;5:1411. doi: 10.1038/s42003-022-04352-2 (PMC9789135; doi:10.1038/s42003-022-04352-2)
Supplement: Supplementary file 3 — Description of Additional Supplementary Files [file 42003_2022_4352_MOESM3_ESM.pdf]

Description of Additional Supplementary Files

File name: Supplementary Data 1

Description: The source data behind the bar plots presented in Figure 2.

File name: Supplementary Data 2

Description: The source data behind the bar plots presented in Figure 3.

File name: Supplementary Data 3

Description: The source data behind the heat plot presented in Figure 4.
