# Supplementary material for: A molecular barcode and web-based data analysis tool to identify imported Plasmodium vivax malaria
Source: Commun Biol. 2022 Dec 23;5:1411. doi: 10.1038/s42003-022-04352-2 (PMC9789135; doi:10.1038/s42003-022-04352-2)
Supplement: Supplementary file 7 — Reporting Summary [file 42003_2022_4352_MOESM7_ESM.pdf]

nature portfolio

Corresponding author(s): A/Prof Sarah Auburn

Last updated by author(s): Nov 23, 2022

Reporting Summary

Nature Portfolio wishes to improve the reproducibility of the work that we publish. This form provides structure for consistency and transparency in reporting. For further information on Nature Portfolio policies, see our Editorial Policies and the Editorial Policy Checklist.

Statistics

For all statistical analyses, confirm that the following items are present in the figure legend, table legend, main text, or Methods section.

n/a	Confirmed	
☐	☒	The exact sample size (*n*) for each experimental group/condition, given as a discrete number and unit of measurement
☐	☒	A statement on whether measurements were taken from distinct samples or whether the same sample was measured repeatedly
☐	☒	The statistical test(s) used AND whether they are one- or two-sided<br>*Only common tests should be described solely by name; describe more complex techniques in the Methods section.*
☐	☒	A description of all covariates tested
☐	☒	A description of any assumptions or corrections, such as tests of normality and adjustment for multiple comparisons
☐	☒	A full description of the statistical parameters including central tendency (e.g. means) or other basic estimates (e.g. regression coefficient) AND variation (e.g. standard deviation) or associated estimates of uncertainty (e.g. confidence intervals)
☒	☐	For null hypothesis testing, the test statistic (e.g. *F*, *t*, *r*) with confidence intervals, effect sizes, degrees of freedom and *P* value noted<br>*Give P values as exact values whenever suitable.*
☐	☒	For Bayesian analysis, information on the choice of priors and Markov chain Monte Carlo settings
☐	☒	For hierarchical and complex designs, identification of the appropriate level for tests and full reporting of outcomes
☐	☒	Estimates of effect sizes (e.g. Cohen's *d*, Pearson's *r*), indicating how they were calculated

Our web collection on statistics for biologists contains articles on many of the points above.

Software and code

Policy information about availability of computer code

Data collection	N/A
Data analysis	All custom, in-house scripts used for data filtering, analyses and visualization are available from https://github.com/vivaxgen/geo. The VivaxGEN-geo web service is accessible at http://geo.vivaxgen.org/.

For manuscripts utilizing custom algorithms or software that are central to the research but not yet described in published literature, software must be made available to editors and reviewers. We strongly encourage code deposition in a community repository (e.g. GitHub). See the Nature Portfolio guidelines for submitting code & software for further information.

Data

Policy information about availability of data

All manuscripts must include a data availability statement. This statement should provide the following information, where applicable:

- Accession codes, unique identifiers, or web links for publicly available datasets
- A description of any restrictions on data availability
- For clinical datasets or third party data, please ensure that the statement adheres to our policy

The study used genomic data on P. vivax derived from the Malaria Genomic Epidemiology (MalariaGEN) P. vivax Genome Variation Project release 4 (Pv4), which has recently been published in Wellcome open Research as an open dataset: https://pubmed.ncbi.nlm.nih.gov/35651694/. This open dataset provide ENA accessions for all samples.

Human research participants

Policy information about studies involving human research participants and Sex and Gender in Research.

Reporting on sex and gender	N/A
Population characteristics	N/A
Recruitment	N/A
Ethics oversight	As described in the published open access data note (https://pubmed.ncbi.nlm.nih.gov/35651694/), the P. vivax samples were all collected from patients with informed consent provided by the patient or a parent or guardian for individuals less than 18 years of age. All studies were approved by local ethics boards as detailed in the data note: All samples in this study were derived from blood samples obtained from patients with P. vivax malaria, collected with informed consent from the patient or a parent or guardian. At each location, sample collection was approved by the appropriate local and institutional ethics committees. The following local and institutional committees gave ethical approval for the partner studies: Human Research Ethics Committee, Walter and Eliza Hall Institute, Australia; Human Research Ethics Committee of NT Department of Health and Families and Menzies School of Health Research, Darwin, Australia; Islamic Republic of Afghanistan Ministry of Public Health Institutional Review Board, Afghanistan; ICDDR,B Ethical Review Committee, Bhutan; Research Ethics Board of Health, at the Ministry of Health in Bhutan; Institutional Review Board of the Institute of Biomedical Sciences, University of São Paulo, Brazil; National Ethics Committee for Health Research, Phnom Penh, Cambodia; Institutional Review Board of Jiangsu Institute of Parasitic Diseases, Wuxi, China; Comite Instiucional de Etica de Investigaciones en Humanos, Colombia; Comite de Bioetica Instituo de Investigaciones Medicas Facultad de Medicina Universidad de Antioquia, Colombia; Armauer Hansen Research Institute Institutional Review Board, Ethiopia; Addis Ababa University College of Natural Sciences, Ethiopia; Addis Ababa University, Aklilu Lemma Institute of Pathobiology Institutional Review Board, Ethiopia; National Research Ethics Review Committee of Ethiopia; Eijkman Institute Research Ethics Committee, Jakarta, Indonesia; Comité National d'Ethique auprès du Ministère de la Santé Publique, Madagascar; National Ethics Committee for Health Research, Lao Peoples' Democratic Republic; Research Review Committee of the Institute for Medical Research and the Medical Research Ethics Committee (MREC), Ministry of Health, Malaysia; The Government of the Republic of the Union of Myanmar, Ministry of Health, Department of Medical Research, Lower Myanmar, Myanmar; Papua New Guinea Institute of Medical Research Institutional Review Board, the Medical Research Advisory Committee of Papua New Guinea; Ethics Review Committee, Faculty of Medicine, University of Colombo, Sri Lanka; Ethics Committee, Faculty of Tropical Medicine, Mahidol University, Bangkok, Thailand; Oxford Tropical Research Ethics Committee, Oxford, UK; Institutional Review Board, National Institute of Allergy and Infectious Diseases, Bethesda, Maryland, USA; Scientific and Ethical Committee of the Hospital for Tropical Diseases in Ho Chi Minh City, Vietnam; The Ministry of Health Evaluation Committee on Ethics in Biomedical Research, Vietnam.

Note that full information on the approval of the study protocol must also be provided in the manuscript.

Field-specific reporting

Please select the one below that is the best fit for your research. If you are not sure, read the appropriate sections before making your selection.

☒ Life sciences ☐ Behavioural & social sciences ☐ Ecological, evolutionary & environmental sciences

For a reference copy of the document with all sections, see nature.com/documents/nr-reporting-summary-flat.pdf

Life sciences study design

All studies must disclose on these points even when the disclosure is negative.

Sample size	The study used a total of 799 P. vivax genomes from 21 countries to establish frameworks for SNP selection and classification of the country of origin of P. vivax infections. A separate set of 142 P. vivax samples was used for independent evaluation of the performance of the classifier in determining country of origin.
Data exclusions	Samples from countries with less than 4 representative P. vivax genomes were excluded from the SNP selection and country classification framework.
Replication	The likelihood-based classification framework was trained on the set of 799 P. vivax genomes, and evaluated in an independent set of 142 P. vivax genomes.
Randomization	N/A
Blinding	N/A

Behavioural & social sciences study design

All studies must disclose on these points even when the disclosure is negative.

Study description	N/A
Research sample	N/A
Sampling strategy	N/A
Data collection	N/A
Timing	N/A
Data exclusions	N/A
Non-participation	N/A
Randomization	N/A

Ecological, evolutionary & environmental sciences study design

All studies must disclose on these points even when the disclosure is negative.

Study description	N/A
Research sample	N/A
Sampling strategy	N/A
Data collection	N/A
Timing and spatial scale	N/A
Data exclusions	N/A
Reproducibility	N/A
Randomization	N/A
Blinding	N/A

Did the study involve field work? ☐ Yes ☒ No

Field work, collection and transport

Field conditions	N/A
Location	N/A
Access & import/export	N/A
Disturbance	N/A

Reporting for specific materials, systems and methods

We require information from authors about some types of materials, experimental systems and methods used in many studies. Here, indicate whether each material, system or method listed is relevant to your study. If you are not sure if a list item applies to your research, read the appropriate section before selecting a response.

Materials & experimental systems

n/a	Involved in the study
☒	☐ Antibodies
☒	☐ Eukaryotic cell lines
☒	☐ Palaeontology and archaeology
☒	☐ Animals and other organisms
☒	☐ Clinical data
☒	☐ Dual use research of concern

Methods

n/a	Involved in the study
☒	☐ ChIP-seq
☒	☐ Flow cytometry
☒	☐ MRI-based neuroimaging

Antibodies

Antibodies used	N/A
Validation	N/A

Eukaryotic cell lines

Policy information about cell lines and Sex and Gender in Research

Cell line source(s)	N/A
Authentication	N/A
Mycoplasma contamination	N/A
Commonly misidentified lines (See ICLAC register)	N/A

Palaeontology and Archaeology

Specimen provenance	N/A
Specimen deposition	N/A
Dating methods	N/A

☐ Tick this box to confirm that the raw and calibrated dates are available in the paper or in Supplementary Information.

Ethics oversight	N/A

Note that full information on the approval of the study protocol must also be provided in the manuscript.

Animals and other research organisms

Policy information about studies involving animals; ARRIVE guidelines recommended for reporting animal research, and Sex and Gender in Research

Laboratory animals	N/A
Wild animals	N/A
Reporting on sex	N/A
Field-collected samples	N/A
Ethics oversight	N/A

Note that full information on the approval of the study protocol must also be provided in the manuscript.

Clinical data

Policy information about clinical studies

All manuscripts should comply with the ICMJE guidelines for publication of clinical research and a completed CONSORT checklist must be included with all submissions.

Clinical trial registration	N/A
Study protocol	N/A
Data collection	N/A
Outcomes	N/A

Dual use research of concern

Policy information about dual use research of concern

Hazards

Could the accidental, deliberate or reckless misuse of agents or technologies generated in the work, or the application of information presented in the manuscript, pose a threat to:

No	Yes	
☒	☐	Public health
☒	☐	National security
☒	☐	Crops and/or livestock
☒	☐	Ecosystems
☒	☐	Any other significant area

Experiments of concern

Does the work involve any of these experiments of concern:

No	Yes	
☒	☐	Demonstrate how to render a vaccine ineffective
☒	☐	Confer resistance to therapeutically useful antibiotics or antiviral agents
☒	☐	Enhance the virulence of a pathogen or render a nonpathogen virulent
☒	☐	Increase transmissibility of a pathogen
☒	☐	Alter the host range of a pathogen
☒	☐	Enable evasion of diagnostic/detection modalities
☒	☐	Enable the weaponization of a biological agent or toxin
☒	☐	Any other potentially harmful combination of experiments and agents

ChIP-seq

Data deposition

☐ Confirm that both raw and final processed data have been deposited in a public database such as GEO.

☐ Confirm that you have deposited or provided access to graph files (e.g. BED files) for the called peaks.

Data access links<br>*May remain private before publication.*	N/A
Files in database submission	N/A
Genome browser session<br>(e.g. UCSC)	N/A

Methodology

Replicates	N/A
Sequencing depth	N/A

Antibodies	N/A
Peak calling parameters	N/A
Data quality	N/A
Software	N/A

Flow Cytometry

Plots

Confirm that:

- ☐ The axis labels state the marker and fluorochrome used (e.g. CD4-FITC).
- ☐ The axis scales are clearly visible. Include numbers along axes only for bottom left plot of group (a 'group' is an analysis of identical markers).
- ☐ All plots are contour plots with outliers or pseudocolor plots.
- ☐ A numerical value for number of cells or percentage (with statistics) is provided.

Methodology

Sample preparation	N/A
Instrument	N/A
Software	N/A
Cell population abundance	N/A
Gating strategy	N/A

☐ Tick this box to confirm that a figure exemplifying the gating strategy is provided in the Supplementary Information.

Magnetic resonance imaging

Experimental design

Design type	N/A
Design specifications	N/A
Behavioral performance measures	N/A

Acquisition

Imaging type(s)	N/A
Field strength	N/A
Sequence & imaging parameters	N/A
Area of acquisition	N/A

Diffusion MRI ☐ Used ☐ Not used

Preprocessing

Preprocessing software	N/A
Normalization	N/A
Normalization template	N/A
Noise and artifact removal	N/A
Volume censoring	N/A

Statistical modeling & inference

Model type and settings	N/A
Effect(s) tested	N/A

Specify type of analysis: ☐ Whole brain ☐ ROI-based ☐ Both

Statistic type for inference (See Eklund et al. 2016)	N/A
Correction	N/A

Models & analysis

n/a	Involved in the study
☐	☐ Functional and/or effective connectivity
☐	☐ Graph analysis
☐	☐ Multivariate modeling or predictive analysis

Functional and/or effective connectivity	N/A
Graph analysis	N/A
Multivariate modeling and predictive analysis	N/A
